# Diffuse Alveolar Hemorrhage Complicating Influenza A Infection in an Immunocompetent Infant: A Case Report with Focused Pediatric Review

**DOI:** 10.3390/jcm15083062

**Published:** 2026-04-17

**Authors:** Hai Thien Do, Hung Trong Dinh, Vuong Minh Tran, Lam Van Nguyen, Tung Viet Cao, Ngoc Nu Hoang Tran

**Affiliations:** 1 Center for Tropical Diseases, The National Children’s Hospital, 18/879 La Thanh, Hanoi 100000, Vietnam; dothienhai.vn@gmail.com (H.T.D.); hungdt.nch@gmail.com (H.T.D.); vuongtm@nch.gov.vn (V.M.T.); lamnv@nch.gov.vn (L.V.N.); 2 The National Children’s Hospital, 18/879 La Thanh, Hanoi 100000, Vietnam; tungcv@nch.gov.vn; 3 College of Health Sciences, VinUniversity, Vinhomes Ocean Park, Gia Lam, Hanoi 100000, Vietnam

**Keywords:** diffuse alveolar hemorrhage, influenza A, infant, pulmonary hemorrhage

## Abstract

**Background**: Influenza is a common cause of hospitalization in young children, particularly infants. While most infections are self-limited, severe and life-threatening complications may occur. Diffuse alveolar hemorrhage (DAH) is a rare pulmonary manifestation of influenza, predominantly reported in adults, and is exceedingly uncommon in immunocompetent infants. **Case Presentation**: We report the case of an 8-month-old previously healthy female infant who presented with influenza A infection and rapidly progressed to acute respiratory failure and shock despite antiviral therapy. Bleeding was noted from the nasal cavity prior to clinical deterioration, and during emergent endotracheal intubation, blood was observed flooding the bronchial tree, consistent with massive pulmonary hemorrhage. Flexible bronchoscopy showed diffusely erythematous and friable airway mucosa without an identifiable focal bleeding source, and early bronchoalveolar lavage was nondiagnostic. Nasopharyngeal testing confirmed influenza A (H3). Laboratory findings revealed severe systemic inflammation, leukopenia with neutropenia, and anemia with normal coagulation parameters. Chest imaging demonstrated bilateral pulmonary infiltrates. After exclusion of autoimmune, coagulation, immunodeficiency, and alternative infectious causes, a diagnosis of diffuse alveolar hemorrhage secondary to influenza A infection was established. The patient was successfully managed with supportive care, antiviral therapy, tranexamic acid, and empiric antibiotics, without corticosteroid treatment, and made a full recovery. **Conclusions**: This case emphasizes that influenza-associated DAH in infants may occur without overt hemoptysis and may not demonstrate classical BAL findings early in the disease course. Clinicians should maintain a high index of suspicion in rapidly deteriorating infants with influenza and diffuse pulmonary infiltrates. The optimal role of corticosteroids remains uncertain and should be individualized.

## 1. Introduction

Influenza is a common acute viral respiratory infection and an important cause of morbidity in children, with infants—particularly those younger than 6 months—experiencing the highest rates of hospitalization and severe disease [1]. Although most pediatric influenza infections are self-limited, serious complications such as pneumonia, acute respiratory distress syndrome (ARDS), and neurologic manifestations may occur, especially in young infants [1].

Diffuse alveolar hemorrhage (DAH) is a rare but life-threatening pulmonary condition characterized by bleeding from the pulmonary microcirculation into the alveolar spaces, leading to acute respiratory failure, anemia, and diffuse pulmonary infiltrates [2]. Pulmonary hemorrhage has been described as a rare complication of influenza, particularly during historical pandemics and in severe infections caused by influenza A (H1N1) and avian influenza A (H5N1); however, it remains uncommon in contemporary pediatric practice [2,3]. Among immunocompetent patients, influenza A is a recognized infectious cause of diffuse alveolar hemorrhage (DAH). However, the overwhelming majority of reported cases occur in adults, and well-documented cases in immunocompetent infants are extremely rare in the available pediatric literature [2].

The pathogenesis of influenza-associated DAH is multifactorial and includes direct viral injury to respiratory epithelium and pulmonary endothelium, disruption of the alveolar–capillary barrier, and inflammation-mediated tissue damage [4,5]. Influenza virus-induced alteration of epithelial tight junctions increases alveolar permeability, resulting in protein-rich edema, fibrin deposition, and hemorrhage [5,6]. Experimental studies also suggest that tissue factor-dependent activation of coagulation pathways plays a protective role in limiting alveolar bleeding during influenza infection, with deficiency associated with increased pulmonary hemorrhage [7].

While respiratory infections are recognized triggers of alveolar hemorrhage episodes in children with underlying DAH conditions, primary influenza-induced DAH in previously healthy infants is rarely reported [8]. Data on its incidence and clinical features in pediatric populations remain limited and are largely derived from isolated case reports rather than systematic studies [2,3]. Reporting such cases is therefore important to improve clinical recognition, facilitate earlier diagnosis, and enhance understanding of this potentially life-threatening but underrecognized complication of influenza infection in infants.

## 2. Case Presentation

An 8-month-old previously healthy female infant was admitted during the winter season with a 2-day history of cough, hoarseness, rhinorrhea, and intermittent fever (5–6 episodes per day), with a maximum recorded temperature of 39.4 °C. She had initially been evaluated at a local clinic, where a rapid antigen test for influenza A was positive, and she was diagnosed with acute nasopharyngitis and left-sided acute otitis media. Outpatient treatment with oseltamivir, ceftibuten, and ibuprofen was initiated; however, her symptoms progressively worsened.

On hospital day 2 (four days after initial symptom onset), the patient experienced abrupt clinical deterioration with acute respiratory distress, marked by severe dyspnea, perioral cyanosis, and blood-tinged nasal and oral secretions, prompting hospital admission. On physical examination, she was tachypneic (64 breaths/min) with diffuse crackles and wheezing on lung auscultation. Peripheral oxygen saturation was 85–90% on room air, improving to 95–98% with supplemental oxygen at 5 L/min via face mask. She was febrile (38.5 °C), alert, and responsive to antipyretic therapy.

Within the same hospital day, her clinical condition rapidly deteriorated, progressing to severe respiratory failure and shock, manifested by tachycardia, hypotension, prolonged capillary refill time (>3 s), and generalized cyanosis (oxygen saturation 75–80%). She was emergently intubated, during which a large volume of bright red blood was suctioned from the endotracheal tube. Mechanical ventilation was initiated using synchronized intermittent mandatory ventilation (SIMV) mode, and vasopressor support with epinephrine was started. Fluid resuscitation was initiated prior to vasopressor support. A diagnosis of pulmonary hemorrhage was made, and intravenous tranexamic acid was administered to control ongoing bleeding.

Initial laboratory investigations revealed marked systemic inflammation and cytopenia, including a C-reactive protein (CRP) level of 100.25 mg/L, ferritin 195 ng/mL, markedly elevated interleukin-6 (2134 pg/mL), and NT-proBNP 1120 pmol/L. Complete blood count revealed leukopenia (WBC 2.74 × 10^9^/L) with neutropenia (16.0%) and relative lymphocytosis (71.7%), normocytic anemia (RBC 3.39 × 10^12^/L; hemoglobin 87 g/L; hematocrit 27.7%), and a normal platelet count (223 × 10^9^/L). Coagulation studies were within normal limits (Prothrombin time 11.2 s, INR 0.97, aPTT 38.3 s, fibrinogen 4.13 g/L). Kidney and liver function tests showed normal results.

**Figure 1:**
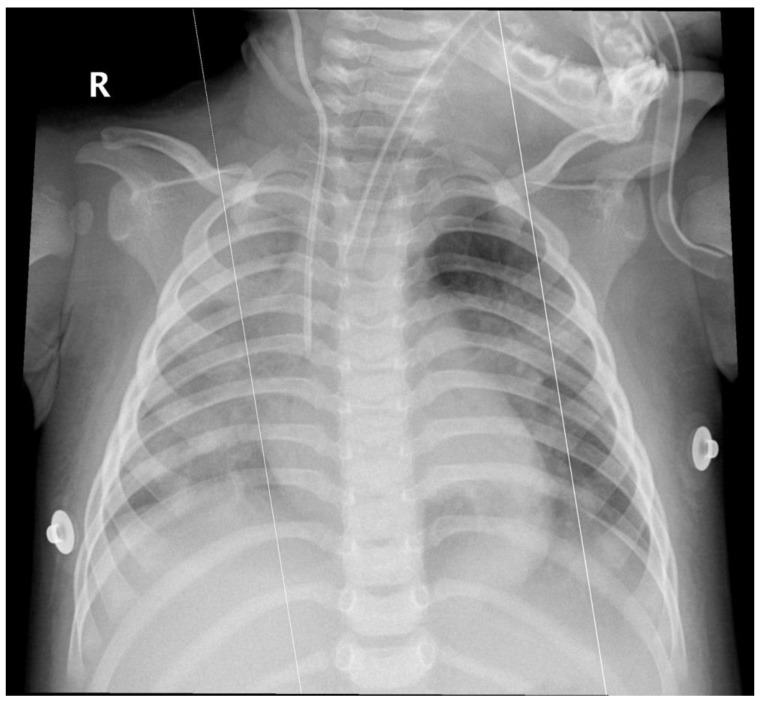
Chest X-ray obtained after intubation, showing diffuse bilateral pulmonary infiltrates.

Arterial blood gas analysis showed acute hypoxemic respiratory failure with respiratory alkalosis (pH 7.49, PaCO_2_ 37 mmHg, PaO_2_ 58 mmHg, HCO_3_^−^ 28.2 mmol/L, lactate 0.6 mmol/L). Chest radiography revealed diffuse infiltrates involving the right lung and the left lower lobe (Figure 1). A nasopharyngeal swab tested positive for influenza A, with subsequent subtype identification confirming influenza A (H3).

**Figure 2:**
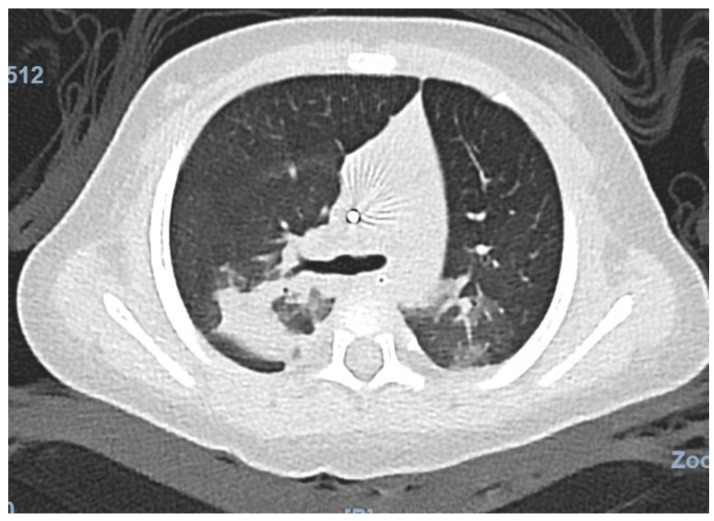
Chest computed tomography revealing bilateral pulmonary infiltrates.

Chest computed tomography demonstrated bilateral pulmonary infiltrates involving the left lower and right upper lobes, as well as a right lower lobe pulmonary nodule, without evidence of pulmonary embolism (Figure 2). Multislice computed tomography angiography did not identify a focal source of bleeding. Flexible bronchoscopy demonstrated diffusely erythematous and friable bronchial mucosa without an identifiable bleeding point. Bronchoalveolar lavage (BAL) fluid cytology revealed a nonspecific inflammatory pattern (total cell count of 1445 cells/µL) without hemosiderin-laden macrophages or malignant cells. Cell block analysis was negative. The bronchoscopy was performed early in the disease course, which may account for the absence of hemosiderin-laden macrophages. Broad microbiological testing of BAL fluid by polymerase chain reaction, including bacterial, mycobacterial, *Pneumocystis jirovecii* and other non-influenza viral pathogens, was negative, and galactomannan antigen testing was also negative.

An extensive evaluation for immunodeficiency and autoimmune disease was unremarkable. Antinuclear antibodies, anti-glomerular basement membrane antibodies, and antineutrophil cytoplasmic antibodies (p-ANCA and c-ANCA) were negative. Urinalysis was normal. Complement levels (C3, C4), serum immunoglobulins (IgG, IgA, IgM, IgE), antiphospholipid antibodies (IgG and IgM), lymphocyte subset analysis (CD3, CD4, CD8, CD19, CD56), and HIV testing were all within normal limits, confirming immunocompetence.

In addition to ongoing antiviral therapy with oseltamivir (3 mg/kg per dose, administered twice daily for a total of 5 days), empiric broad-spectrum antibiotics consisting of meropenem and vancomycin (20 mg/kg per dose, administered three times daily) were given for 15 days to provide coverage for possible secondary bacterial infection in the setting of severe viral pneumonia. With supportive care, the patient’s clinical condition progressively improved.

**Figure 3:**
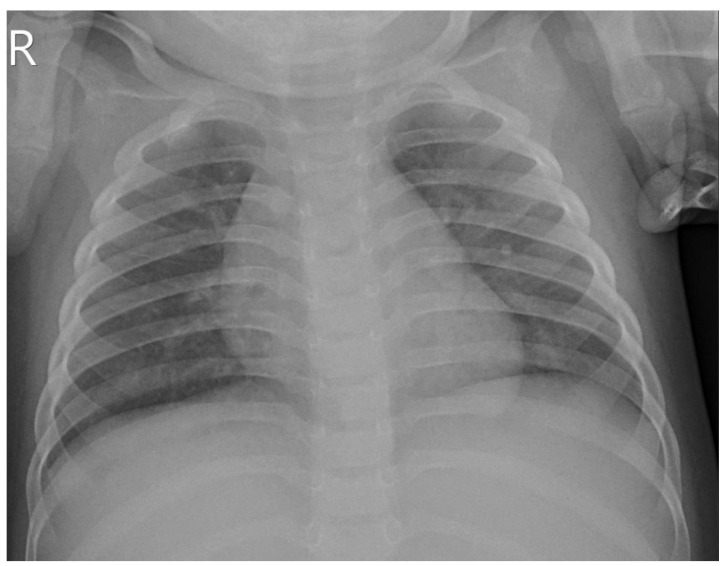
Chest radiograph at discharge demonstrating resolution of pulmonary infiltrates.

After 15 days of treatment, inflammatory markers markedly decreased, with C-reactive protein declining to 6.15 mg/L and interleukin-6 to 1.85 pg/mL, while all other inflammatory parameters and hematologic indices returned to within normal ranges. She was discharged without recurrence of pulmonary bleeding, and follow-up chest radiography demonstrated resolution of pulmonary infiltrates (Figure 3).

Although hemosiderin-laden macrophages were not identified in the initial bronchoalveolar lavage (BAL), the diagnosis of diffuse alveolar hemorrhage was supported by the acute onset of hypoxemic respiratory failure, massive pulmonary bleeding observed during intubation, rapidly progressive bilateral pulmonary infiltrates, unexplained anemia, and bronchoscopic findings of diffusely erythematous and friable airway mucosa without an identifiable focal bleeding source. Bronchoscopy was performed early in the clinical course, and extensive investigations excluded alternative causes of diffuse alveolar hemorrhage, including autoimmune vasculitis, anti-glomerular basement membrane disease, coagulopathy, immunodeficiency, and infectious etiologies other than influenza A. The temporal sequence of clinical events and diagnostic investigations is summarized in Table 1.

## 3. Discussion

### 3.1. Diffuse Alveolar Hemorrhage

Diffuse alveolar hemorrhage (DAH) is a rare but life-threatening pulmonary complication characterized by bleeding from the pulmonary microcirculation into alveolar spaces. In children, DAH typically presents with acute respiratory distress, worsening anemia, and diffuse pulmonary infiltrates; however, hemoptysis may be absent, particularly in infants who lack the ability to expectorate, making early recognition challenging [8,9]. Diagnosis is generally supported by bronchoscopic findings and bronchoalveolar lavage, although classical features may not always be evident at initial presentation.

Etiologically, DAH in pediatric populations may result from immune-mediated disorders, cardiovascular disease, coagulopathies, infections, or idiopathic causes [10,11,12,13,14]. Infections represent an important non-immune trigger in children, and respiratory pathogens have been implicated in episodes of infection-associated alveolar hemorrhage.

A case series of five infants with DAH reported that all patients presented with hemoptysis, mild to severe dyspnea, anemia, and abnormal chest radiographs at the time of diagnosis, with confirmation achieved by bronchoscopy [10]. Identified infectious etiologies in this series included *Chlamydia trachomatis* and Human coronavirus NL63 [10]. In addition, a recent retrospective single-center study described respiratory infection-induced episodes of alveolar hemorrhage in children with DAH, implicating a broad range of pathogens, including *Mycoplasma pneumoniae*, coronaviruses, *Haemophilus influenzae*, *Chlamydia pneumoniae*, *Human Metapneumovirus*, *Acinetobacter baumannii*, *Pneumocystis carinii*, and mixed infections [8]. Although influenza-associated DAH has been described predominantly in adults, reports in immunocompetent infants remain scarce [15,16]. This case highlights that influenza A should be considered in the differential diagnosis of acute pulmonary hemorrhage in infants, even in previously healthy and immunocompetent patients.

### 3.2. Pathophysiology of Influenza-Associated DAH

The pathogenesis of influenza-associated DAH is likely multifactorial, involving direct viral cytopathic effects, dysregulated host inflammatory responses, and disruption of pulmonary hemostatic mechanisms. Influenza A virus can infect and replicate within respiratory epithelial cells throughout the airway, resulting in diffuse alveolar damage accompanied by pulmonary edema and hemorrhage [4]. In addition, the virus has been shown to infect pulmonary microvascular endothelial cells, leading to endothelial injury, disruption of the alveolar–capillary barrier, and subsequent alveolar bleeding [5,6,17]. These processes are mediated in part by apoptosis-related endothelial damage and degradation of tight junction proteins, particularly claudin-5, which are essential for maintaining vascular barrier integrity [17].

Tissue factor (TF) expression on lung epithelial cells plays a critical role in maintaining hemostasis during influenza infection. Experimental studies in murine models have shown that deficiency of TF, particularly within lung epithelial cells, is associated with increased alveolar hemorrhage and higher mortality following influenza A virus infection, indicating that TF-dependent activation of coagulation pathways is necessary to limit pulmonary bleeding [7]. In parallel, the host inflammatory response further aggravates alveolar injury through recruitment of neutrophils and macrophages, release of proinflammatory cytokines such as interleukin-6, interleukin-8, and tumor necrosis factor-α, and dysregulated activation of coagulation pathways, collectively promoting vascular permeability and hemorrhage [18,19,20]. This process may be amplified by a hyperinflammatory state, often described as a “cytokine storm,” which is increasingly recognized in severe influenza [20].

Other respiratory viruses, including respiratory syncytial virus (RSV) and severe acute respiratory syndrome coronavirus 2 (SARS-CoV-2), can also cause acute lung injury through endothelial dysfunction and increased vascular permeability. In SARS-CoV-2 infection, direct viral invasion of endothelial cells and endothelialitis contribute to microvascular thrombosis, capillary leak, and diffuse alveolar damage, with a predominance of thrombotic rather than hemorrhagic complications [21]. In contrast, RSV exhibits a primary tropism for respiratory epithelial cells, and while the resulting inflammatory response is robust, pulmonary hemorrhage is a rare clinical sequela [22]. Influenza viruses, however, may exhibit a distinct propensity for pulmonary vascular injury, partly due to their ability to infect both epithelial and endothelial cells and through the actions of viral surface proteins such as hemagglutinin and neuraminidase, which facilitate host cell entry and viral spread [5,17].

In the present case, the markedly elevated interleukin-6 (IL-6) level (2134 pg/mL) reflects an exaggerated systemic inflammatory response, which may contribute to endothelial dysfunction, increased capillary permeability, and diffuse alveolar damage [20]. This cytokine-mediated injury likely acts synergistically with direct viral cytopathic effects and dysregulated coagulation pathways, ultimately predisposing to diffuse alveolar hemorrhage.

Together, these interconnected mechanisms provide a coherent pathophysiological basis for the development of DAH in severe influenza, particularly in the setting of marked systemic inflammation as observed in this patient.

### 3.3. Diagnostic Approach

The diagnosis of diffuse alveolar hemorrhage is based on a combination of clinical presentation, radiologic findings, and bronchoscopic evaluation with bronchoalveolar lavage (BAL). Classically, DAH is confirmed by bronchoscopy demonstrating progressively bloodier BAL returns and the presence of hemosiderin-laden macrophages, which is considered the diagnostic gold standard [9,23]. However, these findings may be absent early in the disease course, particularly in infants and young children in whom hemoptysis is often unrecognized and bronchoscopy is frequently performed soon after symptom onset.

Imaging typically reveals diffuse alveolar infiltrates, ground-glass opacities, and areas of consolidation, reflecting acute alveolar injury and hemorrhage [8,24]. Given the broad differential diagnosis, a comprehensive evaluation is essential and should include assessment for immune-mediated diseases (such as systemic vasculitis and connective tissue disorders), cardiovascular conditions, coagulopathies, infectious etiologies, and, in infants, consideration of non-accidental trauma [10,25]. In the present case, extensive investigations excluded alternative causes of DAH, supporting the diagnosis of influenza-associated DAH despite the absence of hemosiderin-laden macrophages on early BAL. Therefore, absence of hemosiderin-laden macrophages in early BAL should not exclude DAH when clinical and radiologic findings strongly suggest the diagnosis.

### 3.4. Treatment Strategies

Management of influenza-associated diffuse alveolar hemorrhage involves both etiology-directed therapy and supportive care. Systemic glucocorticoids are frequently used in respiratory infection-induced episodes of alveolar hemorrhage, and one pediatric series reported clinical resolution in 18 of 19 cases following corticosteroid therapy [8]. High-dose methylprednisolone (1–2 mg/kg every 6 h) has been commonly administered in acute severe presentations [26,27]. However, evidence supporting corticosteroid use in influenza-associated DAH remains limited, and optimal treatment strategies have not been established, particularly in infants.

Antiviral therapy with oseltamivir should be initiated promptly in cases of influenza-associated DAH, even when presentation occurs more than 48 h after symptom onset, as treatment may still provide benefit in severe disease [1]. The American Academy of Pediatrics recommends oseltamivir as the antiviral agent of choice for treatment of influenza in children, including hospitalized patients, and supports its use in infants from birth [1,26,28].

Emerging therapies for pediatric DAH include inhaled tranexamic acid, which has shown promise with complete cessation of bleeding in 95% of critically ill children within 48 h in retrospective studies [29]. Intrapulmonary administration of recombinant activated factor VII has also been described as a potential therapeutic option in pediatric DAH, with reported effectiveness and acceptable safety profiles, although evidence remains limited and further controlled studies are required [30]. Supportive management remains fundamental and includes airway protection, correction of anemia and coagulopathy, and careful ventilatory strategies aimed at minimizing ventilator-induced lung injury [9,30].

### 3.5. Prognosis and Outcomes

Diffuse alveolar hemorrhage is associated with substantial mortality, with reported in-hospital mortality exceeding 20% overall [9,31]. Clinical outcomes vary widely depending on underlying etiology and patient population; pediatric cohorts have reported mortality rates of up to approximately 37%, with even higher mortality observed in specific subgroups, particularly those with cardiovascular causes [32]. Early recognition and a systematic diagnostic approach are therefore critical for optimizing clinical outcomes in patients with DAH [9,32]. Long-term follow-up is also essential, as DAH in infants may evolve into a chronic condition characterized by persistent pulmonary abnormalities and incomplete radiologic or functional resolution, and recurrent episodes of pulmonary hemorrhage have been documented in pediatric populations [33,34].

## 4. Conclusions

This case highlights influenza A infection as a rare but potentially life-threatening cause of diffuse alveolar hemorrhage in immunocompetent infants. In this age group, DAH may occur without overt hemoptysis due to limited expectoration, with pulmonary bleeding manifesting only as blood-tinged nasal or oral secretions or becoming apparent after airway instrumentation; moreover, early bronchoalveolar lavage may not reveal hemosiderin-laden macrophages. The rarity of pediatric influenza-associated DAH underscores the need for heightened clinical awareness, prompt diagnostic evaluation, and early initiation of antiviral therapy. Early recognition of influenza-associated DAH is critical, as delayed diagnosis may result in catastrophic respiratory failure. Although the role of corticosteroids in infection-associated DAH remains controversial, this case suggests that favorable outcomes may be achievable with timely antiviral therapy and optimized supportive care alone in some patients. Further studies are warranted to identify clinical and biological predictors of response in infants with influenza-associated DAH, particularly to delineate which patients may improve with supportive care alone versus those who may benefit from adjunctive corticosteroid therapy.

## Figures and Tables

**Table 1:** Timeline of clinical course and diagnostic investigations.

Timepoint	Clinical Events	Investigations
Day 0	Symptom onset (fever, cough)	—
Day 2 (pre-admission)	Worsening symptoms	Influenza A rapid test (+)
Hospital day 2 (admission)	Acute respiratory distress	Chest X-ray
Same day (hours later)	Rapid deterioration → intubation	—
Immediately post-intubation	Mechanical ventilation	CT scan
Early post-intubation (same day)	Critical care	Bronchoscopy with BAL
Hospital days 2–15	Stabilization	Labs, follow-up imaging

## Data Availability

The original contributions presented in this study are included in the article. Further inquiries can be directed to the corresponding author.

## Abbreviations

The following abbreviations are used in this manuscript:

DAH	Diffuse alveolar hemorrhage
CT	Computed tomography
WBC	White blood cells
BAL	Bronchoalveolar lavage
ANA	Antinuclear antibodies
ANCA	Antineutrophil cytoplasmic antibodies
CRP	C-reactive protein
NT-proBNP	N-terminal pro-B-type natriuretic peptide
ARDS	Acute respiratory distress syndrome
HIV	Human immunodeficiency virus
SIMV	Synchronized intermittent mandatory ventilation
INR	International normalized ratio
aPTT	Activated partial thromboplastin time
bpm	Beats per minute
TF	Tissue factor
RSV	Respiratory syncytial virus
SARS-CoV-2	Severe acute respiratory syndrome coronavirus 2
